# 5-Nitrouracil stabilizes the plasma concentration values of 5-FU in colorectal cancer patients receiving capecitabine

**DOI:** 10.1038/s41598-020-59648-2

**Published:** 2020-02-17

**Authors:** Yoichiro Yoshida, Yasuhiro Hashimoto, Makoto Miyazaki, Naoya Aisu, Teppei Yamada, Ryuji Kajitani, Taro Munechika, Yoshiko Matsumoto, Hideki Nagano, Hideki Shimaoka, Akira Komono, Ryohei Sakamoto, Gumpei Yoshimatsu, Fumihiro Yoshimura, Fumiaki Kiyomi, Suguru Hasegawa

**Affiliations:** 1 Department of Gastroenterological Surgery, Fukuoka University Faculty of Medicine, 7-45-1 Nanakuma, Jonan-ku, Fukuoka 814-0180 Japan; 2 FALCO Biosystems Ltd, 346, Shimizu-cho, Nijo-agaru, Kawaramachi-dori Nakagyo-ku, Kyoto, 604-0911 Japan; 3 Academia, Industry and Government Collaborative Research Institute of Translational Medicine for Life Innovation, Fukuoka University, 7-45-1 Nanakuma, Jonan-ku, Fukuoka 814-0180 Japan

**Keywords:** Chemotherapy, Colon cancer

## Abstract

Capecitabine is selectively converted from 5′-DFUR to 5-fluorouracil (5-FU) in tumours by thymidine phosphorylase (TP). We investigated the addition of 5-nitrouracil (5-NU), a TP inhibitor, into blood samples for precise measurements of plasma 5-FU concentrations. The plasma concentration of 5-FU was measured after capecitabine administration. Two samples were obtained at 1 or 2 h after capecitabine administration and 5-NU was added to one of each pair. Samples were stored at room temperature or 4 °C and 5-FU concentrations were measured immediately or 1.5 or 3 h later. The mean plasma 5-FU concentration was significantly higher at room temperature than at 4 °C (*p* < 0.001). The 5-FU concentration was significantly increased in the absence of 5-NU than in the presence of 5-NU (*p* < 0.001). The 5-FU change in concentration was greater in the absence of 5-NU, and reached 190% of the maximum compared with baseline. A significant interaction was found between temperature and 5-NU conditions (*p* < 0.001). Differences between the presence or absence of 5-NU were greater at room temperature than under refrigerated conditions. 5-FU plasma concentrations after capecitabine administration varied with time, temperature, and the presence or absence of 5-NU. This indicates that plasma concentrations of 5-FU change dependent on storage conditions after blood collection.

## Introduction

In current daily practice, the administrated dose of 5-fluorouracil (5-FU) is generally calculated based on the body surface area (BSA). However, BSA has been reported to be a poor predictor of systemic drug exposure^1–3^. 5-FU is characterized by a narrow therapeutic window and strong exposure-toxicity relationship, which support the use of approaches to monitor drug administration. Several investigations have demonstrated a relationship between response and drug exposure in terms of toxicity and efficacy^4,5^. Adjusting the 5-FU dose based on pharmacokinetic monitoring in patients led to a significantly improved response rate and fewer adverse events compared with patients treated with conventional 5-FU dose^6,7^.

Capecitabine, an oral drug that is tumour-selective fluoropyrimidines, is a key pro-drug of 5-FU used in colorectal cancer treatment^8^. Capecitabine is primarily metabolized to 5′-deoxy-5-fluorocytidine (5′-DFCR) by carboxylesterase in the liver^9^; 5′-DFCR is converted to 5′-deoxy-5-fluorouridine (5′-DFUR) by cytidine deaminase, which is predominantly present in the liver as well as in tumour tissues. In the final step, 5′-DFUR is converted to its active form, 5-FU, by thymidine phosphorylase (TP), which is present at higher concentrations in cancer than in normal tissues^10^. Phase I trials have demonstrated a relationship between the occurrence of adverse events and the exposure to capecitabine metabolites^11,12^. Although Cmax and area under the curve (AUC) for 5′-DFUR and α-fluoro-β-alanine were found to be predictive of dose-limiting toxicities, systemic exposure to 5-FU was poorly predicted. Furthermore, Gieschke reported that the plasma concentrations of 5-FU, 5′-DFUR and α-fluoro-β-alanine do not necessarily reflect concentrations in normal tissues and cancers after capecitabine therapy^13^.

Incurred sample reanalysis (ISR) is the confirmation of the reproducibility of quantitative values by re-measurement of blood samples collected after the administration of test drugs to animals and humans^14^. The acceptance criteria for ISR data are advocated by the bioanalytical community and should be within 20% of the original sample results for nonligand-binding small-molecule assays^15^. However, ISR analysis for 5-FU in samples from patients that were dosed orally with capecitabine failed this evaluation while 5-FU intravenously dosed patients in the same study had acceptable ISR results^16^. The experimental evidence indicated that 5′-DFUR conversion to 5-FU was the primary cause for ISR failure.

5-Nitrouracil (5-NU) is similar in structure to 5-FU and inhibits TP, blocking the reaction from 5′-DFUR to 5-FU^17^. Because capecitabine is predominantly converted to 5-FU in tumours, 5-FU levels in blood are typically measured after the oral administration of capecitabine without a metabolic inhibitor such as 5-NU. However, it was reported that 5′-DFUR continued to be converted to 5-FU even after samples had been stored at −70 °C^16^. Here we analysed changes in 5-FU plasma concentration by using blood samples with the addition of 5-NU, which inhibits the conversion of 5′-DFUR to 5-FU.

## Results

**Figure 1:**
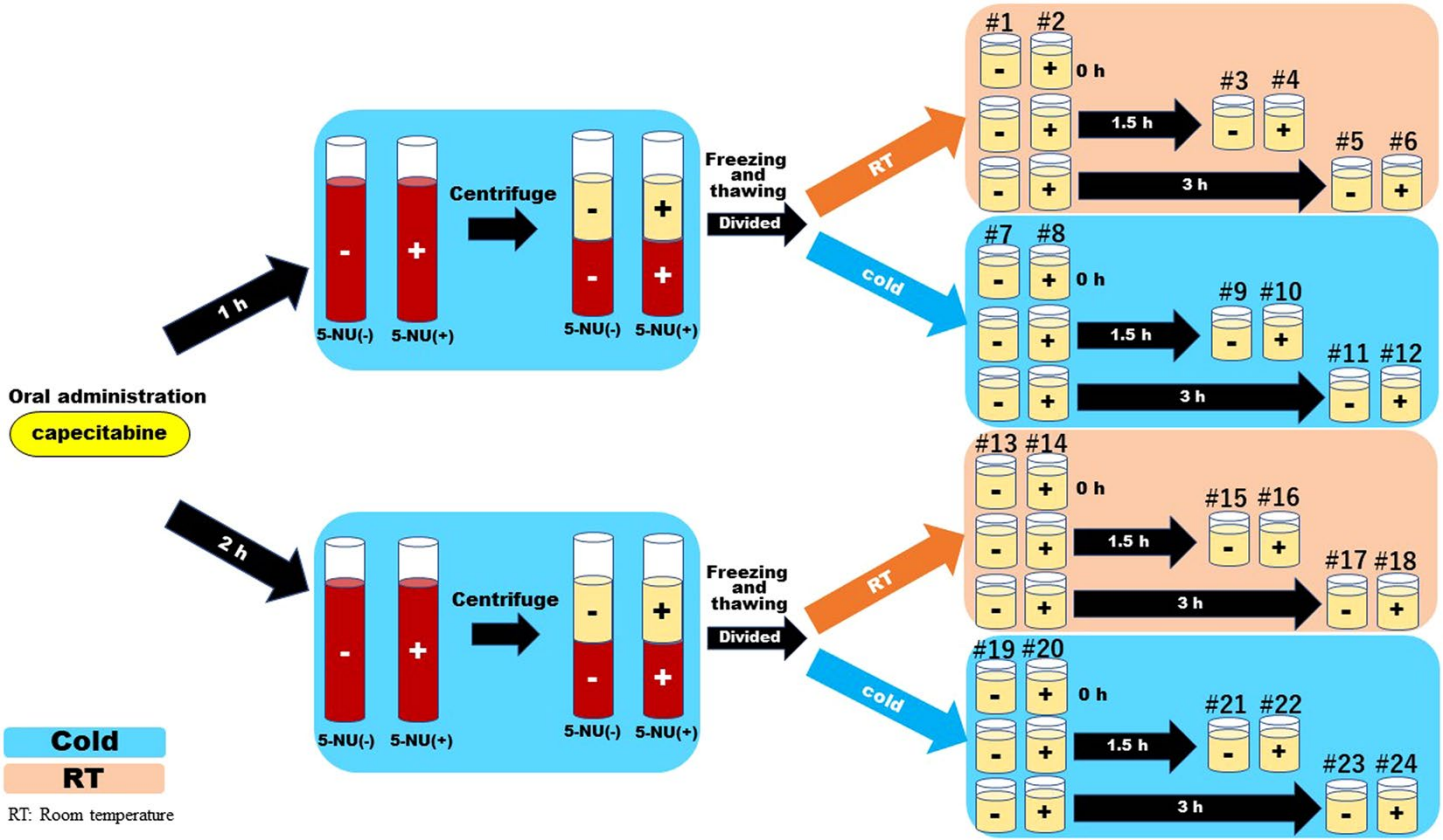
Measurement schedule of blood concentrations of 5-FU in colorectal cancer patient samples. Blood samples were obtained from colorectal cancer patients (n = 6) after the first capecitabine administration. The plasma concentration of 5-FU was measured under 24 different conditions to investigate the effect of temperature, time, and 5-NU on 5-FU concentration. Blood samples (5 ml) were collected at 1 h and 2 h after the administration of capecitabine (1000 mg/m^2^) into two EDTA blood collection tubes for each time point. To one of the two tubes, 5-NU was added. The samples were centrifuged at 4 °C and the plasma component was stored at −80 °C. After freezing and thawing, each sample was divided in half with one half placed at room temperature (room) and the other placed on ice (cold). The 5-FU plasma concentration was measured at three time points: immediately, after 1.5 h and after 3 h.

**Figure 2:**
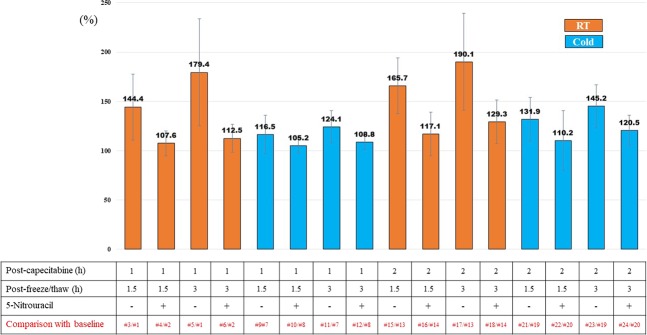
Changes of 5-FU plasma concentrations compared with baseline (%). The 5-FU plasma concentration immediately after thawing (baseline) was designated 100%. Data represent the mean and the bars represent the standard deviation. ^#^The number used in Fig. 1.

We collected samples under 24 conditions as shown in Fig. 1, and the 5-FU plasma concentrations are shown in Table 1. The mean blood 5-FU concentration was significantly higher at room temperature storage than at 4 °C, irrespective of the presence of 5-NU, and the difference increased over time. In the absence of 5-NU, the concentration of 5-FU was significantly increased under all conditions over time. The plasma concentration of 5-FU immediately after thawing at room temperature was 119.7 ng/ml (# 1) but increased to 228.6 ng/ml (# 5) after 3 h. The concentration after 3 h was only increased to 136.6 ng/ml after adding 5-NU. When measured at 4 °C, the concentration after 3 h was reduced to 145.1 ng/ml (# 11). The change of 5-FU plasma concentration compared with the baseline value determined immediately after thawing is shown in Fig. 2. The change of plasma 5-FU concentrations was the highest in samples obtained 2 h after capecitabine followed by storage at room temperature without 5-NU, but was minimal in the sample obtained 1 h after capecitabine followed by storage under refrigerated conditions. Larger differences in 5-FU measurements were detected in the absence of 5-NU, with a maximum difference of 190%. The addition of 5-NU and measurement at 4 °C suppressed the increase in plasma concentration of 5-FU over time.

**Table 1:** Plasma concentration of 5-FU (ng/ml).

Time after capecitabine (h)	Temperature	Time after freezing and thawing (h)	5-Nitrouracil	
			(−)	(+)
1	RT (25 °C)	0	#1: 119.7 (78.4)	#2: 126.2 (63.9)
		1.5	#3: 182.5 (154.9)	#4: 132.2 (63.4)
		3	#5: 228.6 (218.3)	#6: 136.6 (62.3)
	Cold (4 °C)	0	#7: 119.7 (78.3)	#8: 126.2 (63.9)
		1.5	#9: 134.4 (76.8)	#10: 130.9 (62.3)
		3	#11: 145.1 (87.1)	#12: 133.7 (61.4)
2	RT (25 °C)	0	#13: 68.8 (41.6)	#14: 76.9 (30.0)
		1.5	#15: 106.6 (55.1)	#16: 90.4 (39.3)
		3	#17: 122.3 (66.5)	#18: 97.0 (34.5)
	Cold (4 °C)	0	#19: 68.8 (41.6)	#20: 76.9 (30.1)
		1.5	#21: 85.7 (46.3)	#22: 87.1 (46.3)
		3	#23: 94.7 (49.9)	#24: 91.8 (35.2)

Data represent the mean (standard deviation).

Numbers correspond with sample numbering shown in Fig. 1.

RT: Room temperature.

Table 2 shows the analysis of the percentage change in plasma 5-FU concentrations from the baseline (immediately after freezing and thawing) using a mixed effect model including time from capecitabine administration, temperature, 5-NU, and time from thawing as fixed effects and using patients as a random effect. A statistically significant difference was observed for all conditions: time after oral administration (1 h vs 2 h, *p* = 0.001), temperature (room temperature vs 4 °C, *p* < 0.001), 5-NU (present vs absent, *p* < 0.001), and the time after freezing and thawing (1.5 h vs 3 h, *p* = 0.01). The factor that showed the largest change was the presence or absence of 5-NU. A significant interaction was noted between the temperature and 5-NU conditions (*p* < 0.001) (Table 3). The difference between the presence and absence of 5-NU was greater at room temperature than under refrigerated conditions. Given the significant interaction between temperature and 5-NU, a subset analysis was performed for room temperature and refrigerated storage conditions. A significant difference was observed between the presence and absence of 5-NU as a stabilizing agent both at room temperature storage and at refrigerated storage.

**Table 2:** Analysis of 5-FU plasma concentration (ng/ml).

Factor	Estimated mean		Difference (95% CI)	P-value
Time after oral administration	1 h	2 h		
	124.8	138.8	−13.9 (−24.5, −3.4)	0.001
Temperature	RT	Cold		
	143.3	120.3	23.0 (12.5, 33.5)	<0.001
5-NU	(−)	(+)		
	149.7	113.9	35.8 (25.3, 46.3)	<0.001
Time after freezing and thawing	1.5 h	3 h		
	124.8	138.7	−13.9 (−24.4, −3.4)	0.010

The percentage change from baseline (immediately after freezing and thawing) was assessed using a mixed effect model including time from capecitabine administration, temperature, 5-NU, and time from thawing as fixed effects and the patient as a random effect.

RT: Room temperature.

**Table 3:** Interactions between 5-NU and other factors.

Interaction		Estimated mean		P-value
5-NU:Time after oral administration		1 h	2 h	0.523
	5-NU (−)	141.1	158.2	
	5-NU (+)	108.5	119.2	
5-NU:Time after freezing and thawing		1.5 h	3 h	0.219
	5-NU (−)	139.6	159.7	
	5-NU (+)	110.0	117.8	
5-NU:Temperature		RT	Cold	<0.001
	5-NU (−)	169.9	129.4	
	5-NU (+)	116.6	111.2	

The percentage change from baseline (immediately after freezing and thawing) was assessed using a mixed effect model including time from capecitabine administration, temperature, 5-NU, time from thawing, and interactions between 5-NU and the other three effects as fixed effects and the patient as a random effect.

RT: Room temperature.

## Discussion

The efficacy of 5-FU therapeutic drug monitoring has been validated in several studies, which demonstrated the significant superiority of pharmacokinetic monitoring compared with conventional 5-FU dose to improve response rates and decrease severe toxicity^6,18^. Therefore, a lot of research has been conducted to assess an appropriate dose adjustment algorithm^7,19^. The AUC of 5-FU plasma concentrations appears to be the most relevant pharmacokinetic parameter associated with 5-FU-related toxicity and efficacy. Several studies proposed a 5-FU target AUC of 20–24 mg·h/L^6,20^. Therefore, accurate measurement of plasma 5-FU concentration is critical with such a narrow therapeutic window.

Capecitabine is selectively converted to 5-FU in cancers via a cascade of three enzymes: carboxyl esterase, cytidine deaminase, and TP^10^. When measuring 5-FU blood concentrations after the administration of capecitabine, inhibitors of these three enzymes are not included in the blood samples because capecitabine is not thought to be converted to 5-FU until it reaches the tumour. Therefore, no previous study has attempted to measure blood 5-FU concentrations in the presence of these enzyme inhibitors following oral capecitabine administration. TP converts 5′-DFUR to 5-FU and also functions as an angiogenic factor^21,22^. TP is expressed at high levels in glial cells, macrophages, stromal cells, and some epithelia^23^. A high correlation was found between tumour TP levels and serumTP levels (r = 0.65, *p* < 0.0001)^24^. However, serum TP levels were considerably lower compared with tumour TP levels^24,25^. Therefore, conversion of 5′-DFUR to 5-FU in blood is considered negligible because of the low biological activity of TP.

**Figure 3:**
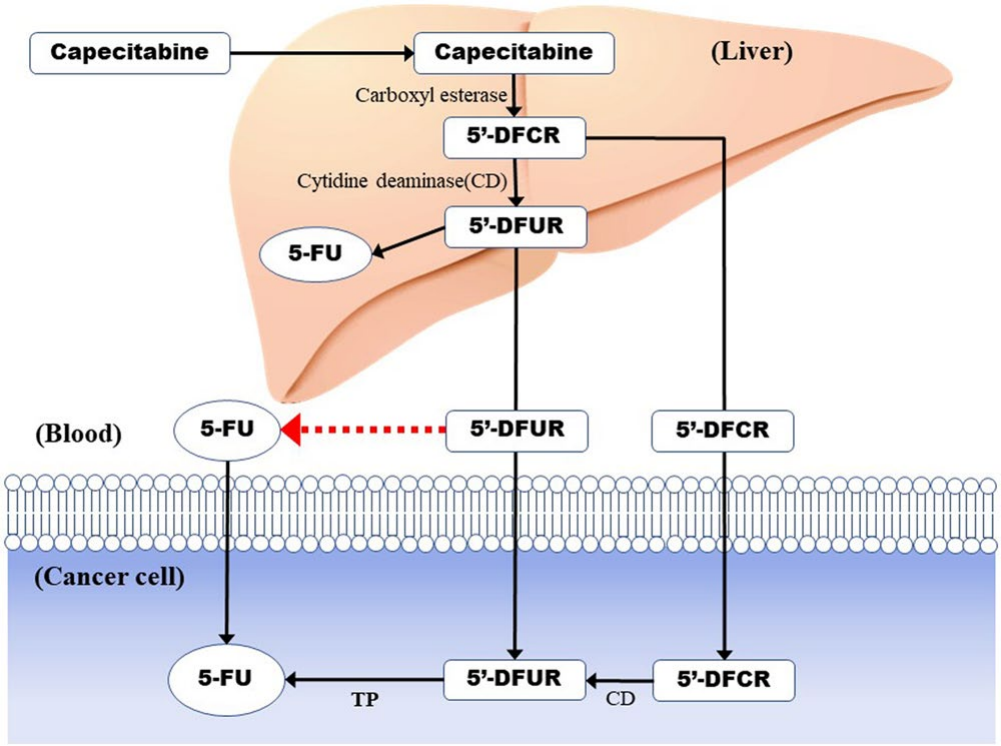
Metabolism of capecitabine. Capecitabine is selectively converted from 5′-DFUR to 5-FU in cancer cells by thymidine phosphorylase (TP). The addition of 5-NU, which is an inhibitor of TP, suppressed the increase in plasma concentration of 5-FU, indicating that 5′-DFUR is converted to 5-FU in the blood. In the absence of TP inhibitor, capecitabine can be converted to 5-FU even in the blood (red arrow).

In this study, we added 5-NU to blood collection tubes and observed chronological changes in the blood concentration of 5-FU following capecitabine administration. Our results showed that the concentration of 5-FU increased nearly two-fold under some measurement conditions. Lowering the measurement temperature and adding 5-NU suppressed the increase in the plasma concentration of 5-FU over time. Therefore, metabolism to 5-FU occurred at room temperature or in the absence of 5-NU. Notably, the addition of 5-NU, an inhibitor of TP, suppressed the elevation of blood concentrations of 5-FU. This indicates that plasma components may contain TP and that 5′-DFUR may be converted to 5-FU in the blood (Fig. 3, red arrow). Gamelin *et al*. examined plasma 5-FU concentrations within the therapeutic range based on past study results and reported that the use of plasma 5-FU concentrations, rather than BSA, as an index to adjust dosage was more conducive to successful treatment with fewer serious adverse events. Measuring 5-FU concentrations in blood was previously difficult because of the need for specialized equipment, but since the development of new kits, relatively simple tests are available. The My5-FU assay is a nanoparticle-based immunoassay that can be run on a standard clinical laboratory automated clinical chemistry analyser, enabling high sample throughput^26^. The My5-FU assay showed equivalent performance on different analysers such as liquid chromatography-tandem mass spectrometry (LC-MS) and other clinical analyzers^27^. A linear relationship (r = 0.9204) exists between My5-FU assays and LC-MS for plasma concentrations of capecitabine^28^. My5-FU allows for the measurement of the plasma concentration of 5-FU, which was conventionally measured using special analysis equipment as well as a general clinical laboratory instrument. Therefore, monitoring of blood levels of anti-cancer drugs, which was previously difficult, has become possible in clinical practice. The My5-FU procedure recommends the use of a DPD inhibitor as a stabilizer. DPD, the catalysing enzyme in the first and rate-limiting steps of the 5-FU degradation process, degrades approximately 85% of administered 5-FU^29^. When measuring blood levels of 5-FU, the addition of DPD inhibitors is common, even when using measuring methods other than My5-FU. However, the inclusion of inhibitors of enzymes involved in converting capecitabine to 5-FU is not common. As shown in this study, 5-FU blood levels after capecitabine administration are dependent on various measurement conditions, such as time, temperature, and the presence or absence of 5-NU. The reason that the difference in 5-FU plasma concentration was the highest at 2 h after capecitabine administration is unknown, and further evaluation is necessary. Although our data show it is preferable to measure plasma 5-FU concentrations at 4 °C immediately after blood collection, this may not be practical in actual clinical settings because of various reasons. In such cases, it is possible to minimize the changes in 5-FU blood concentrations by adding 5-NU. The simplest way to avoid false plasma concentrations of 5-FU is to analyse plasma samples of patients immediately after the collection of whole blood. By using My5-FU, these measurements can be performed easily and quickly. It is desirable to convert this modality for patients who will receive capecitabine irrespective of their clinicopathological factors. However, it is necessary to consider cost-effectiveness because this methodology will increase medical expenses. Because the accurate measurement of plasma 5-FU concentrations is critical not only for reducing and preventing adverse events but also for predicting responses, further investigation is necessary.

There were some limitations in this study. First, the sample size was small. This study had a very efficient design using repeated measurements. This design allows the intra-individual variation to be used as a residual error for statistical analyses. Thus, although the sample size was 6, which is not large, statistically significant differences were obtained. Second, although 5-FU plasma concentrations were measured by antibody reaction, My5-FU can easily measure the plasma concentrations of 5-FU using common clinical laboratory equipment. Third, capecitabine showed high pharmacokinetic variability; therefore, the Time to Maximum Effect (Tmax) of metabolites may occur later than the sampling time (2 h) of capecitabine. Thus, extending the sampling time may cause further differences in plasma concentrations of 5-FU. In addition, this was a pure *in vitro* study without the establishment of an *in vivo* model. To characterize the results properly, it is desirable to establish additional animal models. However, considering that the lethal dose 50 (mouse) of 5-nitrouracil is 3.25 μg/g, it will be difficult to administer these amounts of 5-nitrouracil to an animal model. Although these limitations remain, we have not only demonstrated the instability of the plasma concentration of 5-FU after capecitabine administration, but also suggested the need for inhibitors such as TP.

## Patients and Methods

### Patients and eligibility criteria

Six patients treated with capecitabine for colorectal cancer were enrolled in this study between June and September 2017. This study was performed in accordance with the ethical guidelines for clinical studies. The institutional review board at Fukuoka University approved the protocol (16-10-02). Informed consent was obtained from all patients prior to study entry. All procedures were performed in accordance with the Declaration of Helsinki.

Eligible patients were ≥20 years of age, with histologically confirmed colorectal cancer without prior chemotherapy and radiotherapy for metastatic cancer. The patients also met the following criteria: life expectancy ≥3 months; Eastern Cooperative Oncology Group performance status 0–1; neutrophil count ≥1000/mm^3^; haemoglobin ≥8.0 g/dL; platelet count ≥75000/mm^3^; serum creatinine ≤1.5 times the upper normal limit; and total bilirubin ≤2.0 mg/dl.

Patients were excluded on any one of the following conditions: serious drug allergy; severe peripheral neuropathy; active infection; uncontrollable hypertension; mechanical or paralytic bowel obstruction; uncontrollable diabetes mellitus; cirrhosis; unstable ischemic heart disease; multiple malignancy within the last 5 years; ascites or pleural effusion or pericardial effusion; uncontrolled diarrhoea; and any other criteria making a patient unsuitable for this study.

### Measurement of 5-FU plasma concentration

The overall study flow for sample collection is shown in Fig. 1. We evaluated the plasma concentration of 5-FU in 24 different conditions per patient. Blood samples (5 ml) were collected at 1 h and 2 h after administration of capecitabine (1000 mg/m^2^) into two EDTA blood collection tubes for each time point. To one of the two tubes, 100 μL of 15 mM 5-NU was added. The samples were centrifuged at 4 °C and the plasma component was aspirated and stored at −80 °C until analysis. After freezing and thawing, each plasma sample was divided in half with one half placed at room temperature and the other placed on ice. The 5-FU plasma concentration was measured at three time points: immediately, after 1.5 h and after 3 h. We measured the concentrations twice for each condition and calculated the mean value. The concentrations of 5-FU were measured by photometric detection using a homogeneous competitive nanoparticle immunoassay (My5-FU; Saladax Biomedical, Bethlehem, PA, USA) and analysed on a commercial Abbott Architect C4000 biochemical analyser as described^26–28,30–33^. Saladax provides a stabilizer kit as a dihydropyrimidine dehydrogenase (DPD) inhibitor. The assay is based on the aggregation of nanoparticles that is inversely proportional to the amount of 5-FU in the sample.

### Statistical analyses

The percentage change in plasma 5-FU concentration from the baseline (immediately after freezing and thawing) was assessed using the mixed effect model with the time elapsed after capecitabine administration (1 and 2 h), temperature (room temperature/refrigeration), presence/absence of 5-NU, and time elapsed after freezing and thawing (1.5 and 3 h) as the fixed effects and the patient as a random effect. Interactions between 5-NU and the other three effects were also evaluated. The sample size was not statistically calculated because this was an exploratory study. We decided to measure 24 points for each of the six patients. A P value less than 0.05 was considered statistically significant. Data were analysed using SAS version 9.4 (SAS Institute, Cary, NC, USA).

## Data Availability

The data that support the findings of this study are available from the corresponding author upon reasonable request.

## References

[1] Felici A, Verweij J, Sparreboom A (2002). Dosing strategies for anticancer drugs: the good, the bad and body-surface area. European journal of cancer (Oxford, England: 1990).

[2] Saif MW, Choma A, Salamone SJ, Chu E (2009). Pharmacokinetically guided dose adjustment of 5-fluorouracil: a rational approach to improving therapeutic outcomes. Journal of the National Cancer Institute.

[3] Gamelin E, Boisdron-Celle M (1999). Dose monitoring of 5-fluorouracil in patients with colorectal or head and neck cancer–status of the art. Critical reviews in oncology/hematology.

[4] Milano G (1994). Relationship between fluorouracil systemic exposure and tumor response and patient survival. Journal of clinical oncology: official journal of the American Society of Clinical Oncology.

[5] Di Paolo A (2008). 5-fluorouracil pharmacokinetics predicts disease-free survival in patients administered adjuvant chemotherapy for colorectal cancer. Clinical cancer research: an official journal of the American Association for Cancer Research.

[6] Gamelin E (2008). Individual fluorouracil dose adjustment based on pharmacokinetic follow-up compared with conventional dosage: results of a multicenter randomized trial of patients with metastatic colorectal cancer. Journal of clinical oncology: official journal of the American Society of Clinical Oncology.

[7] Capitain O (2012). Individual fluorouracil dose adjustment in FOLFOX based on pharmacokinetic follow-up compared with conventional body-area-surface dosing: a phase II, proof-of-concept study. Clinical colorectal cancer.

[8] Saif MW, Katirtzoglou NA, Syrigos KN (2008). Capecitabine: an overview of the side effects and their management. Anti-cancer drugs.

[9] Walko CM, Lindley C (2005). Capecitabine: a review. Clinical therapeutics.

[10] Miwa M (1998). Design of a novel oral fluoropyrimidine carbamate, capecitabine, which generates 5-fluorouracil selectively in tumours by enzymes concentrated in human liver and cancer tissue. European journal of cancer (Oxford, England: 1990).

[11] Blesch KS (2003). Clinical pharmacokinetic/pharmacodynamic and physiologically based pharmacokinetic modeling in new drug development: the capecitabine experience. Investigational new drugs.

[12] Ackland SP, Peters GJ (1999). Thymidine phosphorylase: its role in sensitivity and resistance to anticancer drugs. Drug resistance updates: reviews and commentaries in antimicrobial and anticancer chemotherapy.

[13] Gieschke R, Burger HU, Reigner B, Blesch KS, Steimer JL (2003). Population pharmacokinetics and concentration-effect relationships of capecitabine metabolites in colorectal cancer patients. British journal of clinical pharmacology.

[14] Rocci ML, Devanarayan V, Haughey DB, Jardieu P (2007). Confirmatory reanalysis of incurred bioanalytical samples. The AAPS journal.

[15] Fast DM (2009). Workshop report and follow-up–AAPS Workshop on current topics in GLP bioanalysis: Assay reproducibility for incurred samples–implications of Crystal City recommendations. The AAPS journal.

[16] McKnight, J. *et al*. In *8th Workshop on Recent Issues in Bioanalysis (WRIB) Universal City*, *California*. 10–14 (2014).

[17] Miszczak-Zaborska E, Wozniak K (1997). The activity of thymidine phosphorylase obtained from human uterine leiomyomas and studied in the presence of pyrimidine derivatives. Zeitschrift fur Naturforschung. C, Journal of biosciences.

[18] Fety R (1998). Clinical impact of pharmacokinetically-guided dose adaptation of 5-fluorouracil: results from a multicentric randomized trial in patients with locally advanced head and neck carcinomas. Clinical cancer research: an official journal of the American Association for Cancer Research.

[19] Kaldate RR, Haregewoin A, Grier CE, Hamilton SA, McLeod HL (2012). Modeling the 5-fluorouracil area under the curve versus dose relationship to develop a pharmacokinetic dosing algorithm for colorectal cancer patients receiving FOLFOX6. The oncologist.

[20] Patel JN (2014). A community-based multicenter trial of pharmacokinetically guided 5-fluorouracil dosing for personalized colorectal cancer therapy. The oncologist.

[21] Moghaddam A (1995). Thymidine phosphorylase is angiogenic and promotes tumor growth. Proceedings of the National Academy of Sciences.

[22] Miyadera K (1995). Role of thymidine phosphorylase activity in the angiogenic effect of platelet-derived endothelial cell growth factor/thymidine phosphorylase. Cancer research.

[23] Fox SB (1995). Platelet-derived endothelial cell growth factor/thymidine phosphorylase expression in normal tissues: an immunohistochemical study. The Journal of pathology.

[24] Katayanagi S (2003). Measurement of serum thymidine phosphorylase levels by highly sensitive enzyme-linked immunosorbent assay in gastric cancer. Oncology reports.

[25] Shimada H (2002). Prognostic significance of serum thymidine phosphorylase concentration in esophageal squamous cell carcinoma. Cancer.

[26] Freeman K (2016). Is monitoring of plasma 5-fluorouracil levels in metastatic/advanced colorectal cancer clinically effective? A systematic review. BMC cancer.

[27] Buchel B (2013). Comparative evaluation of the My5-FU immunoassay and LC-MS/MS in monitoring the 5-fluorouracil plasma levels in cancer patients. Clinical chemistry and laboratory medicine.

[28] Makihara K (2012). A pilot study of pharmacokinetically guided dose management of capecitabine in CRC patients. Journal of Clinical Oncology.

[29] Yoshida Y (2015). 5-Fluorouracil chemotherapy for dihydropyrimidine dehydrogenase-deficient patients: potential of the dose-escalation method. Anticancer research.

[30] Beumer JH (2009). Multicenter evaluation of a novel nanoparticle immunoassay for 5-fluorouracil on the Olympus AU400 analyzer. Therapeutic drug monitoring.

[31] Freeman K (2015). Fluorouracil plasma monitoring: systematic review and economic evaluation of the My5-FU assay for guiding dose adjustment in patients receiving fluorouracil chemotherapy by continuous infusion. Health technology assessment (Winchester, England).

[32] Mindt S (2019). Therapeutic drug monitoring (TDM) of 5-fluorouracil (5-FU): new preanalytic aspects. Clinical chemistry and laboratory medicine.

[33] Iwai T (2018). Capecitabine reverses tumor escape from anti-VEGF through the eliminating CD11bhigh/Gr1high myeloid cells. Oncotarget.

